# Systematic Review and Meta-Analysis of Diabetes Knowledge among Type 2 Diabetes Patients in Southeast Asia

**DOI:** 10.1900/RDS.2021.17.82

**Published:** 2021-10-31

**Authors:** Phei Ching Lim, Retha Rajah, Chong Yew Lee, Te Ying Wong, Sherene Su Ann Tan, Sarah Abdul Karim

**Affiliations:** 1 Hospital Pulau Pinang, Penang, Malaysia,; 2 School of Pharmaceutical Sciences, Universiti Sains Malaysia, Penang, Malaysia.

**Keywords:** type 2 diabetes mellitus, knowledge, Southeast Asia, knowledge factors

## Abstract

**OBJECTIVE:**

Recognition of patient baseline knowledge is important in educating patients with type 2 diabetes mellitus (T2D) to manage their disease effectively. The purpose of this study is to review current evidence on the level of diabetes knowledge among T2D patients and determine factors affecting their knowledge.

**METHODS:**

A systematic search of English language articles published between 1990 and June 2019 was conducted using six electronic databases. Only quantitative studies that assessed knowledge of T2D patients in Southeast Asian countries were included. Data were extracted and a meta-analysis was conducted.

**RESULTS:**

A total of 6210 articles were retrieved; seven articles met the inclusion criteria, comprising 1,749 T2D patients. The calculated mean knowledge score was 55.6% (95% CI: 7.6 to 103.6). Five types of assessment tools were identified ranging from five to 41 questions that focused on disease specifics, treatment, and nutrition. Age, education level, and glycemic control were the most common factors impacting knowledge.

**CONCLUSIONS:**

The level of knowledge among T2D patients in Southeast Asia was unsatisfactory, especially in older patients with low education levels and poor glycemic control. Hence, an appropriate educational plan should be prioritized to these groups.

## 1 Introduction

Type 2 diabetes mellitus (T2D) has emerged as one of the most challenging public health problems of the 21^st^ century. The disease has risen at an alarming rate with a worldwide prevalence of 463 million people in 2019. In developing countries, particularly in Southeast Asia, the incidence rate is much higher following population growth, aging, lifestyle changes, and the increasing prevalence of obesity. Southeast Asia consists of eleven countries, including Vietnam, Thailand, Cambodia, Laos, Myanmar, Malaysia, Singapore, Indonesia, Brunei, the Philippines, and Timor-Leste. According to recent updates in 2019 by the International Diabetes Federation, 29 million people are living with diabetes in these countries. Malaysia has the highest prevalence rate of adults aged more than 20 years that have T2D (16.8%) followed by Singapore (14.2%) and Brunei (13.2%). Indonesia, as the highest populated country in the region, has the highest number of people living with diabetes, 10.7 million. If interventional strategies are not implemented, this number is projected to increase to 46 million by the year 2045 [1].

Besides appropriate usage of antidiabetic medication, effective diabetes management also depends on the patients’ knowledge about their disease, healthy eating options, physical exercise, and self-monitoring of blood glucose levels [2]. Despite various pharmacological modalities available today, T2D prevalence continues to increase. Poor disease knowledge is one of the main reasons for suboptimal self-care behavior and failure to achieve recommended glycemic targets in diabetic patients [3,4]. Poor glycemic control (HbA1c ≥ 7%) may result in microvascular and macrovascular complications such as kidney failure, retinopathy, neuropathy, myocardial infarction, stroke, and peripheral vascular disease, which in turn lead to increased morbidity and mortality as well as economic challenges [5,6].

Although knowledge about the disease alone does not bring about the required behavior modifications to achieve optimal treatment outcomes, the assessment of patient knowledge level is crucial in designing personalized educational interventions tailored to the individual needs of each T2D patient. Diabetes education should aim to improve patients’ knowledge about their disease and self-care behavior to achieve optimal glycemic control and reduction in diabetes-related complications [7]. In order to implement effective and individualized diabetes education, the patients’ knowledge level should be determined first and specific knowledge gaps identified. Recent standards of care recommendations provided by the American Diabetes Association (2019) emphasize patient participation or a “patient-centered” approach in charting the course of disease management together with the healthcare providers [8]. A patient-centered communication style that uses person-centered, strength-based language, and active listening to elicit patient preferences and beliefs, assessment of literacy and numeracy, and identification of potential barriers to diabetes care are recommended. When patients participate in creating the treatment plan, adherence to the treatment regimen is increased. It is therefore necessary for a T2D patient to have a minimum level of basic knowledge to be able to participate in the decision-making process. Baseline knowledge about the disease also enables further enhancement of a patient’s knowledge level through education.

Given the high prevalence of T2D in Southeast Asian countries and the importance of diabetes-related knowledge in achieving positive outcomes, a comprehensive review of the knowledge level of T2D patients in this region is highly warranted to inform diabetes educators in formulating educational interventions with an emphasis on patient participation. To date, only a handful of systematic reviews conducted in Southeast Asia countries involved T2D patients, but none of them specifically provided data on diabetes knowledge. Therefore, the present study aims to identify and examine available literature on the knowledge level of T2D patients in the Southeast Asia region. Also, this review provides data on the types of knowledge assessment tools that were used and the factors affecting the knowledge level.

## 2 Methods

This systematic review was conducted in accordance with the Preferred Reporting Items for Systematic Reviews and Meta-Analyses (PRISMA) recommendations [9] and was registered at the National Medical Research Register of Malaysia (NMRR). The study did not require ethical approval as all analyses were performed based on data extracted from previous published studies.

A comprehensive search of the literature published from 1990 to June 2019 was performed using six electronic databases: CINAHL, Medline, Google Scholar, PubMed, Sage Journals, and Science Direct. The subject terms and keywords used were: ‘diabetes’, ‘knowledge’, ‘comprehension’ and ‘understanding’. In addition, the bibliography of retrieved articles was screened for relevant titles. For example, the PubMed search strategy involved terms and keywords as follows: diabetes [MeSH Term] AND knowledge [All field] OR comprehension [All field] OR understanding [All field].

### 2.1 Review process and study quality assessment

All titles and abstracts of the retrieved articles were screened initially for relevance by one of the investigators (LPC). After removal of duplicates, the retrieved articles were assessed for eligibility by two investigators independently (STSA and WTY). Decisions to include or exclude a study were compared between the two investigators. In case of disagreement and if the primary reviewers could not reach a consensus, the other investigators were consulted to resolve the disagreement.

The following inclusion criteria were applied:

- Articles were published in the English language.
- Studies contain descriptions of disease-related knowledge inT2D patients.
- Studies were conducted in Southeast Asia countries.

The exclusion criteria comprised:

- Review and qualitative study.
- Development and validation of diabetes knowledge questionnaire.
- Type 1 diabetes and gestational diabetes.
- Description of patient perception and attitude.

Subsequently, the investigators (STSA and WTY) retrieved full texts of articles that were identified as potentially relevant. The following study details were extracted and included in the data collection form for each study:

1. Study characteristics, including year and country of publication, study design, sample size, response rate.
2. Demographics of the study population.
3. Assessment tool, number, and type.
4. Knowledge score.
5. Factors associated with knowledge.

Quality assessment of eligible studies was performed independently by two reviewers (RR and SAK) using a set of quality criteria adapted from the method by Loney *et al*.[10]. The tool contains eight questions, including four questions on sample selection, one on comparability, and three on outcome measurement. Meeting all criteria in a category would confer a high score in the category except for the comparability criterion, whereby studies that meet <50% of the criteria would be considered as having a low score.

### 2.2 Statistical analysis

This study focused on reviewing evidence on the knowledge level of T2D patients in Southeast Asia, especially the knowledge score. Descriptive statistics were applied to explore the knowledge score across the data extracted from the studies. In studies lacking the mean percentage of knowledge score, this was calculated by according to the following equation and added:

This meta-analysis was conducted using Microsoft Office Excel 2013 [11]. For continuous variables, we computed the weight of each study and then the effect size. The Q-test was applied and I^2^-values were calculated and used to determine the heterogeneity among the studies. I^2^-values of 25%, 50%, and 75% were considered to represent low, moderate, and considerable heterogeneity, respectively [12,13]. The random effect model was applied because of possible sampling errors and variations in the populations and aspects of knowledge assessed in the studies. The outcome was a mean knowledge score for each study as a percentage of knowledge level among the patients, and the 95% confidence interval was calculated [11]. The possibility of a publication bias was assessed by construction of a funnel plot of standard errors versus the knowledge scores in the logarithmic scale.

## 3 Results and discussion

The initial electronic database search identified a total of 6210 potentially relevant publications. After removal of duplications, titles and abstracts were screened for relevance, resulting in the exclusion of 4165 articles. The remaining 149 articles underwent full-text examination, and seven studies [14-20] were finally included in this review. The reasons for exclusion were:

- The studies were not conducted in Southeast Asian countries (n=98).
- The populations were not T2D patients (n=20).
- The outcomes were not knowledge-related (n=10).

**Figure 1.**
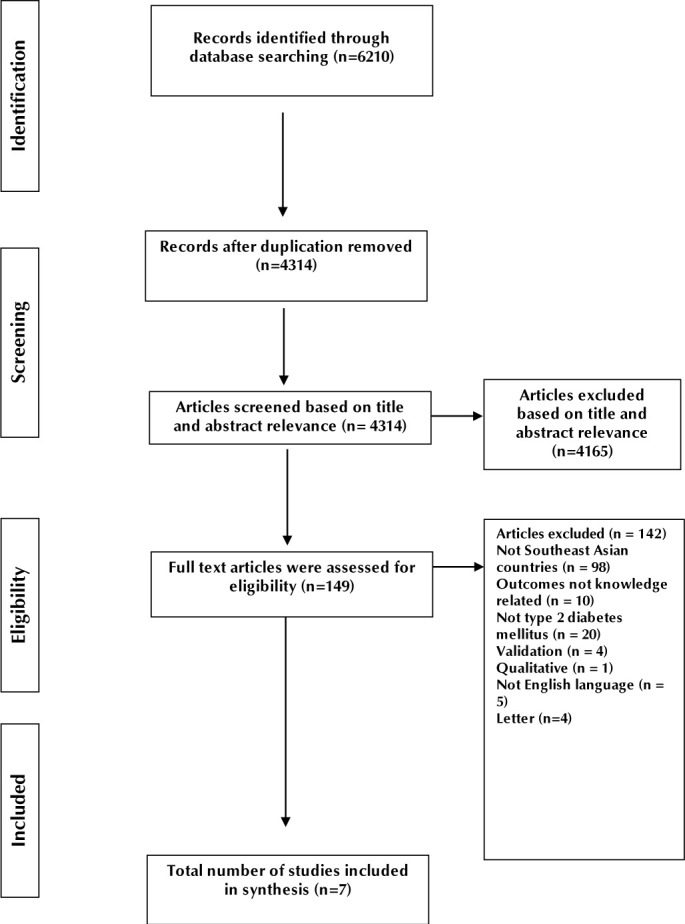
Flow chart for study screening and selection according to PRISMA guidelines.

**Figure 1** illustrates the study screening and selection process as well as the reasons for exclusion of studies.

The studies included were conducted in three out of 11 countries in Southeast Asia: Malaysia, (n = 4)[14,15,17,19], the Philippines, (n = 1) [16], and Thailand, (n = 2) [18,20]. A total of 1749 T2D patients were assessed regarding their knowledge through a cross-sectional survey. The majority of the patients were female (53.7%), and the mean age was 57.1 years (ranging from 53.3 to 62.1 years). The studies were conducted in specialized diabetes clinics (n = 3) [15,19,20] and district area clinics (n = 4) [14,16,17,18], consisting of 984 patients and 765 patients, respectively. The results are summarized in (Table 1).

**Table 1.** Summary of study characteristics, knowledge assessment tools, knowledge score, and factors associated with knowledge

(publication year), country	Sample size, sampling method, response rate (%)	Population characteristics	Assessment tool, number of questions, type of questions (n)	Score	Mean Knowledge Score (%), mean SD), prevalence of good knowledge	Factors associated with knowledge	Findings
Abbasi *et al*. (2018), Malaysia	n=386, convenience sampling, 97%	Mean age 54.7± 7.8 years, 52.6% female, 44.6% had diabetes durationof5 to 10 years	Translated Michigan Diabetes Knowledge Test (MDKT) 14 questions Disease-specific (4), nutrition (6), monitoring (2), foot care (1), exercise (1)	>14 good 12-14 fair <12 poor	58.5 (22.1) 15.5% with good knowledge	Strong: academic qualification, attitude, practice Moderate: income Weak: occupation Very weak: type of therapy, diabetes education, age	Majority of patients had moderate level of knowledge (47.7%)
Al-Qazaz *et al*. (2011), Malaysia	n=505, convenience sampling, 93.5%	Mean age 58.2±9.2 years, 50% male, duration of diabetes of 9.7±6.3 years	Translated and validated MDKT 14 questions Disease-specific (4), nutrition (6), monitoring (2), foot care (1), exercise (1)	>11 good 7-11 average <7 poor	53.1 (-) 11.5% with good knowledge	Younger age, higher educational level, high monthly income, home monitoring, longer duration of diabetes, lower HbA1c were associated with better knowledge score	The knowledge among the patients was inadequate and needs to be improved
Ding *et al*.(2006), Malaysia	n= 83, systematic sampling, 85.6%	Mean age 53.3 years, 60.2% male	Diabetes knowledge questionnaire from Wee *et al*. (2002) and Tham et al. (2004) 41 questions Disease-specific (8), risk factors (4), symptoms and complications (12), treatment (13), monitoring (4)	Total score in percentage	81.8 (10.9)	Having a family member with diabetes was associated with better knowledge	The knowledge of patients with diabetes was classified as acceptable
Kueh *et al*. (2016), Malaysia	n=266, convenience sampling, 100%	Mean age 57.0± 8.5 years, 51.5% female, duration of diabetes 10.4±7.5 years	Diabetes Knowledge Scale (DKN) 15 questions Disease-specific (9), nutrition (6)	Mean knowledge in %	52.5 (17.0)	No association between diabetes knowledge and quality of life	Diabetes knowledge could reduce the impact on quality of life indirectly by influencing other variables such as attitudes
Ardena *et al*. (2010), the Philippines	n=156, stratified cluster sampling, 100%	Mean age 56.7± 10.5 years, 67.3% female, 56.4% had diabetes duration of less than 1 year	Translated and validated American Association of Clinical Endocrinologist (AACE) 24 questions Disease-specific (8), nutrition (4), monitoring (3), exercise (4), treatment (5)	Overall mean % score	42.7 (14.8)	Younger age and highest education attainment scored better	Overall knowledge score was poor
Eknithiset *et al*. (2018), Thailand	n=140, random sampling, 100%	Mean age 62.1± 7.0 years, 69.4% female, duration of diabetes of 13.7± 6.0 years	Validated structured questionnaire Number and type of questions were not stated	Total 7 points. Good knowledge if score > mean	51.6 (-)	Gender	Patients’ knowledge was significantly poor; females had significantly better knowledge
Thewjitcharoen *et al*. (2018), Thailand	n=213, not stated	Mean age 57.4±10.9 years, 52.6% female, median duration of diabetes of 14 years	Validated Theptarin Diabetes questionnaire 5 questions Nutrition (5)	High 5 Moderate 3-4 Low <3	54.0 (-) 6% had high scores	No association between diabetes nutritional knowledge and the actual diabetes self-care behavior	The majority of the patients (55%) scored moderately

Only one study conducted by Ardena *et al*.[16] received a maximum score for the quality assessment, and none of the studies had a low score (Table 2). The participants of three studies were randomly allocated using systematic sampling [17], stratified cluster sampling [16], and random sampling [18], but only two studies described the methods of random sampling adequately [16,17]. Four studies had a low score for selection (score of 0-2), which was due to convenience sampling [14,15,19] and the absence of a description on sample size calculation [20]. Also, four studies included samples from more than one center [14-16,18], while the remaining three studies recruited their samples from one setting only. All studies received high comparability scores as demographic characteristics of the study population were included. Five studies had high scores for outcome (score of 3), while two studies were scored moderately (score of 2) because they failed to provide the reasons why some patients refused to continue their participation [14,15].

**Table 2.** Quality assessment

Study	Selection				Comparability	Outcome			Total score
	Sampling method appro-priate for the research question?	Sample frame appro-priate?	Sample size adequate?	Are the health outcomes measured in an unbiased fashion?	Is the estimation of prevalence or incidence given with confidence interval and in detail by subgroup if appropriate?	Study objectives suitable, and standard criteria used for measurement of the health outcomes?	Are the subjects and the setting described in detail and similar to those of interest to you?	Is the response rate adequate? Are the refusers described?	
Abbasi *et al*. (2018)	X	✓	✓	✓	✓	✓	✓	X	6
Al-Qazaz *et al*. (2011)	X	✓	✓	✓	✓	✓	✓	X	6
Ding *et al*. (2006)	✓	X	X	✓	✓	✓	✓	✓	6
Kueh *et al*. (2016)	X	X	X	✓	✓	✓	✓	✓	5
Ardena *et al*. (2010)	✓	✓	✓	✓	✓	✓	✓	✓	8
Eknithiset *et al*. (2018)	✓	X	X	✓	✓	✓	✓	✓	6
Thewjitcharoen *et al*. (2018)	X	X	✓	✓	✓	✓	✓	✓	6

### 3.1 Knowledge scores and knowledge assessment tools

**Figure 2.**
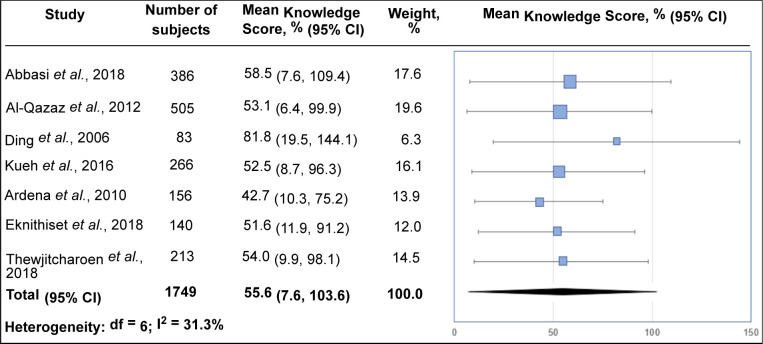
Meta-analysis of mean knowledge scores (%).

The forest plot for the knowledge score is shown in **Figure 2**, and subgroup analysis is summarized in (**Table 3**). The overall mean knowledge score was 55.6% (95% CI: 7.6 to 103.6) with moderate heterogeneity. The knowledge level of patients in specialized diabetes clinics was homogenous and corresponded with the mean knowledge score of 52.8%. In contrast, the knowledge score of patients in district area clinics was rather heterogeneous (I^2^ = 32.8%) as the populations involved three countries, and the studies used different types of knowledge assessment tools. There was no significant difference between the knowledge scores of T2D patients in specialized diabetes and district area clinics. Using the Michigan Diabetes Knowledge Test (MDKT) to assess knowledge [21], the mean knowledge scores were 58.5% and 53.1% in district area clinics [14] and specialized diabetes clinics [15], respectively.

**Table 3.** Subgroup analysis

Study location	Studies (n)	Mean score (95% CI)	I^2^ (%)
Overall	7	55.6 (7.6, 103.6)	31.3
Specialized diabetes clinics	3	52.8 (4.6, 100.9)	0
District areas	4	59.8 (16.3, 103.3)	32.8

A total of 99 questions were identified from five types of knowledge assessment tools which were utilized in the studies. The knowledge assessment tools are shown in (**Table 1**). The questions consisted of 5 to 41 questions that were self-administered by the patients [14,17,19,20] or investigator-administered after face-to-face interview [15,16]. Most of the questions were disease-specific, consisting of 29 questions (29.3%), followed by questions related to nutrition (21.2%) and treatment (18.2%). In MDKT, a question related to foot care was included. Patients’ knowledge in relation to symptoms, complications, and risk factors [22,23] was assessed in a study conducted in district area clinics in Malaysia [17]. A study from Thailand utilized only five out of 10 questions in the Theptarin diabetes questionnaire developed by Thewjitcharoen *et al*. [20]. Only questions related to nutrition were selected as the aim of the study was to assess knowledge related to nutrition among the patients. Other questions were related to physical exercise [21,24] and monitoring of glycemic parameters and the occurrence of adverse events [21-24].

**Figure 3.**
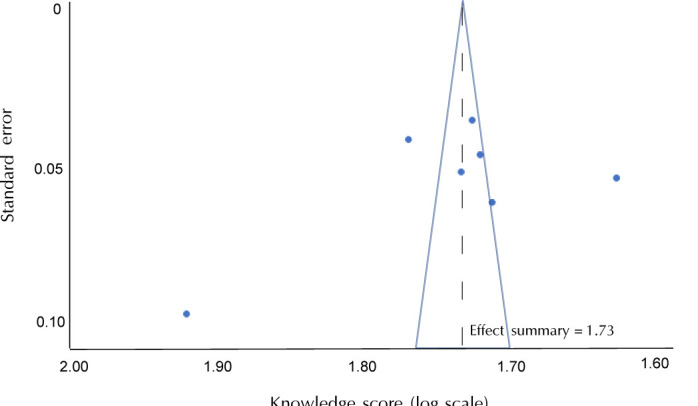
Funnel plot of knowledge scores of the seven studies reviewed to assess the publication bias.

**Figure 3** shows the funnel plot indicating asymmetry of the data. Notably, one study was at the left of the overall estimate due to having the lowest knowledge score [16]. In contrast, the study by Ding *et al*. with the highest mean knowledge score was found at the rightmost side of the overall estimate [17].

### 3.2 Factors associated with knowledge

Five of the seven studies included analyzed demographic characteristics associated with knowledge [14-18]. Younger age and higher level of education were associated with higher knowledge scores [14-16]. Studies that assessed knowledge using MDKT showed that the following patients groups had significantly higher knowledge scores [14, 15]:

1. Patients aged less than 45 years old (p<0.05).
2. Patients with a university education (p<0.001).
3. Patients who have monthly incomes of more than RM2000 (USD 476.0, based on USD 1.0 = RM 4.2) or RM2500 (USD 595) (p<0.001).

Furthermore, the study by Al-Qazaz *et al*. demonstrated that glycosylated hemoglobin (HbA1c) of less than 7% was associated with better knowledge (p<0.001) [15]. Meanwhile, Ardena *et al*. reported that patients aged less than or equal to 60 years (p=0.026) and high school and college graduates had higher knowledge scores (p=0.046) [16]. Patients with family members or relatives with diabetes were found to have better diabetes-related knowledge (83.4% versus 78.2%) [17]. Gender was also associated with knowledge in a study conducted in rural basic healthcare centers of Thailand. None of the studies reported an association between knowledge and duration of diabetes [18].

Studies by Kueh *et al*. [19] and Thewjitcharoen *et al*.[20] did not focus on patient-specific variables associated with knowledge, but emphasized quality of life [19] and diabetes self-care behavior [20]. Both studies concluded that there was no association between knowledge and quality of life or self-care behavior.

## 4 Discussion

The most obvious observation was the very limited number of studies available in Southeast Asian countries that report on diabetes knowledge among T2D patients. Data on diabetes-related knowledge are available from only three out of eleven countries belonging to this region. Furthermore, a few issues even debilitate the quality of the studies, particularly regarding the methodology. The majority of the studies (n = 4) used convenience sampling that may have implications for external validity. Lack of sample size calculation was noted in one study, and some studies were conducted in one center only, which may raise concerns about the representativeness of the target population and have implications for confidence in reported outcomes.

This review highlighted an unsatisfactory level of diabetes knowledge with a wide variation (7.6 % to 103.6%), reflecting substantial discrepancies among the studies mainly due to dissimilarity of the tools used to measure knowledge. Most of the tools were translated from the English language to local or native languages to minimize the risk of language barriers. Although the knowledge tools used were validated, several questions may not be relevant in the setting of Southeast Asian countries. This may be due to the knowledge assessment tools being developed in Western countries such MDKT by Fitzgerald *et al*.[25], Diabetes Knowledge Scale (DKN) by Collins *et al*.[21], and the diabetes knowledge evaluation form by the American Association of Clinical Endocrinologists [26]. For instance, questions pertaining to nutritional intake may not be relevant because of differences in dietary intake among the Southeast Asia populations.

The main contribution of this review lies in investigating factors affecting knowledge of T2D patients in Southeast Asia countries. The findings are crucial to understand fully the variables that influence knowledge in this region and to provide information on which factors are significant and consequently can be targeted for future interventions to raise knowledge levels. Similar to previous systematic reviews on factors affecting patient knowledge [27,28], age, education attainment, and income were factors identified that affect diabetes knowledge. Generally, higher risk of decline in cognitive function, memory, learning, attention, and psychomotor efficiency is a potential barrier to learning in patients with advanced age, whereas younger patients may have more motivation and adaptability to manage their disease [29].

This review also revealed greater knowledge among T2D patients with a higher level of education. Education improves the ability of individuals to access, evaluate, and use information, enabling them to seek valuable health knowledge. Skills that accompany higher educational achievement include positive attitudes towards health based on their awareness regarding prevention and achieving treatment goals. The need to provide simple and precise information especially to elderly (>60 years) cohorts and patients with low education should be emphasized in order to ensure acceptance of treatment goals and management outcomes. The high association between education and income may explain the finding of insufficient knowledge among lower income groups of T2D patients. These patients were also more likely to live in a low-income neighborhood that lacks resources for optimal healthcare.

Another interesting finding was that T2D patients with family members or relatives with diabetes were associated with better diabetes knowledge. A close relative with the chronic disease means that there is a good source of information available to the patient, besides support and encouragement [30]. One of the strategies that can be adopted is the formation of diabetes support groups that keep patients and family members motivated so that they can adhere to their disease management routines and become a source of inspiration to other patients. However, there was no significant difference in patient knowledge scores between diabetes care provided in specialized diabetes clinics attended by specialists and consultants or health clinics in district areas that were attended by medical officers. This suggested that the expertise level of the healthcare provider was not an important determinant of patient knowledge level. As for gender, only one study documented a significant association, namely that females were associated with better knowledge, which was attributed to women having better health information or healthcare-seeking behavior [18]. The finding may suggest establishing gender-specific diabetes programs. However, more studies need to be carried out to support this.

The strength of this review includes the multi-database search strategy using six databases, the involvement of two reviewers at every phase including screening of articles, the assessment of eligibility, and the monitoring quality of included articles. Although extensive search strategies were implemented to identify relevant literature, some studies may have been missed because of the different terminology used to describe diabetes knowledge. The exclusion of non-English language studies also may have reduced the representativeness of our findings. The studies included were cross-sectional in nature and not interventional, which was reflected by their sampling methods. Nevertheless, the studies that used convenience sampling collected sample sizes sufficiently large to provide generalization values. The small number of eligible studies included in the final analysis may explain the large confidence intervals observed. The wide variety of assessment tools employed in this review meant that scoring systems were different. This could have led to inconsistent results across the studies, thus making a comparison of findings between studies challenging. These differences may also explain the asymmetry observed in the funnel plot (Figure 3). The small number of studies included (<10) meant that the power was too low to distinguish a publication bias based on funnel plot asymmetry only.

On the basis of the small number of studies included, this review concludes that the knowledge level of T2D patients was insufficient in Southeast Asia countries in order to prevent or mitigate late complications of diabetes. This suggested a lack of patient education and empowerment. A multidisciplinary approach and the development of diabetes education to improve T2D patient knowledge focusing on different socio demographic and clinical characteristics are highly warranted. Also, future studies should use a standardized validated knowledge assessment tool specially developed for Southeast Asian populations to assess better the knowledge level in T2D patients.

## Abbreviations

T2D type 2 diabetes mellitus

PRISMA Preferred Reporting Items for Systematic Reviews and Meta-Analyses

NMRR National Medical Research Register of Malaysia

MDKT Michigan Diabetes Knowledge Test

DKN Diabetes Knowledge Scale
